# DNMT1-maintained hypermethylation of Krüppel-like factor 5 involves in the progression of clear cell renal cell carcinoma

**DOI:** 10.1038/cddis.2017.323

**Published:** 2017-07-27

**Authors:** Rong-Jie Fu, Wei He, Xiao-Bo Wang, Lei Li, Huan-Bin Zhao, Xiao-Ye Liu, Zhi Pang, Guo-Qiang Chen, Lei Huang, Ke-Wen Zhao

**Affiliations:** 1 Institute of Health Sciences, Shanghai Institutes for Biological Sciences (SIBS), University of Chinese Academy of Sciences, Chinese Academy of Sciences (CAS) & Shanghai Jiao Tong University School of Medicine (SJTU-SM), Shanghai, China; 2 Department of Pathology, Ren-Ji Hospital Affiliated to Shanghai Jiao Tong University School of Medicine, Shanghai, China; 3 Department of Pathophysiology, Key Laboratory of Cell Differentiation and Apoptosis of Chinese Ministry of Education, Shanghai Jiao Tong University School of Medicine (SJTU-SM), Shanghai, China

## Abstract

Clear cell renal cell carcinoma (ccRCC) is the major subtype of renal cell carcinoma (RCC) that is resistant to conventional radiation and chemotherapy. It is a challenge to explore effective therapeutic targets and drugs for this kind of cancer. Transcription factor Krüppel-like factor 5 (KLF5) exerts diverse functions in various tumor types. By analyzing cohorts of the Cancer Genome Atlas (TCGA) data sets, we find that KLF5 expression is suppressed in ccRCC patients and higher level of KLF5 expression is associated with better prognostic outcome. Our further investigations demonstrate that *KLF5* genomic loci are hypermethylated at proximal exon 4 and suppression of DNA methyltransferase 1 (DNMT1) expression by ShRNAs or a methylation inhibitor 5-Aza-CdR can recover KLF5 expression. Meanwhile, there is a negative correlation between expressions of KLF5 and DNMT1 in ccRCC tissues. Ectopic KLF5 expression inhibits ccRCC cell proliferation and migration/invasion *in vitro* and decreases xenograft growth and metastasis *in vivo*. Moreover, 5-Aza-CdR, a chemotherapy drug as DNMTs’ inhibitor that can induce KLF5 expression, suppresses ccRCC cell growth, while knockdown of KLF5 abolishes 5-Aza-CdR-induced growth inhibition. Collectively, our data demonstrate that KLF5 inhibits ccRCC growth as a tumor suppressor and highlight the potential of 5-Aza-CdR to release KLF5 expression as a therapeutic modality for the treatment of ccRCC.

Renal cell carcinoma (RCC), with the high incidence occurring in developed countries, is the most frequent form of kidney cancer.^1^ RCC arises from the proximal renal tubular epithelium of kidneys and accounts for about 85% of renal cancers. Clear cell renal cell carcinoma (ccRCC) is the most common subtype of RCC, accounting for 70–75% of cases.^2^ Owing to the higher expression of multidrug resistance genes, therapeutic options for ccRCC are limited. To date, the main approach for ccRCC is complete or partial nephrectomy combined with chemotherapy and radiotherapy. Other therapies, including immunotherapy with interleukin-2 (IL-2) or interferon alpha (IFN*α*), have been demonstrated low efficiency because of systemic toxicities during ccRCC treatment.^3, 4, 5^ Therefore, further discoveries of effective therapeutic targets and drugs are paramount to improve the prognosis of ccRCC patients.

DNA methylation is an epigenetic process in which adds a methyl group to the cytosine ring at CpG dinucleotides. It has an important role in the regulation of gene expression through interfering with transcriptional factors binding to DNA, recruiting methyl-CpG-binding proteins to repress DNA transcription and affecting histone modifications and chromatin structure. It has been widely investigated that alterations of DNA methylation, especially hypermethylation of tumor suppressor genes, mediate tumorigenesis.^6^ The Cancer Genome Atlas (TCGA) Project has revealed that increasing hypermethylation frequency is correlated with advanced tumor stage in ccRCC.^7^ DNA methylation is mainly facilitated by DNA methyltransferase (DNMT) 1, DNMT3A and DNMT3B.^8^ Li *et al.* demonstrated that DNMT1, DNMT3A and DNMT3B expression were significantly higher in ccRCC tissues compared with non-tumor tissues.^9^ These findings further confirm the important roles of DNA methylation in ccRCC progression. Decitabine (Dacogen), the clinical form of DNMTs inhibitor 5-Aza-2'-deoxycytidine (5-Aza-CdR), has been an approved therapy for the treatment of hematological malignancies, as myelodysplastic syndrome and acute myeloid leukemia (AML). Moreover, decitabine is used in treatment of some solid tumors combined with other drugs.^10, 11, 12^ Hagiware *et al.*^13^ found that 5-Aza-CdR could suppress Caki-1 (a human metastatic RCC cell line) growth *in vivo*. Negrotto *et al.*^14^ illuminated low dose 5-Aza-CdR treatment could be a potential non-cytotoxic therapy for RCC. Hence, epigenetic treatment with 5-Aza-CdR seems to be a promising therapeutic regimen for ccRCC.

Krüppel-like factor 5 (KLF5) belongs to Krüppel-like transcription factors family, of which 17 members have been identified.^15, 16^ KLF5 widely expresses in different tissues and has essential roles in various physiological and pathological processes including cell cycle, angiogenesis, migration, apoptosis, inflammation, self-renew and differentiation.^17^ Notably, it has been reported that KLF5 is overexpressed in some types of human cancers, like breast and bladder cancer, in which it promotes cancer cells proliferation, metastasis and angiogenesis.^18, 19^ Whereas, it has also been demonstrated that KLF5 is deleted or downregulated in other human cancer types such as prostate cancer and AML, in which it inhibits tumor growth and promotes differentiation.^20, 21^ Thus, KLF5 functions as an oncogene or a tumor suppressor due to its cellular and genetic context-dependent regulation of target genes.^22, 23^ In kidney and its collecting system, KLF5 was reported to express in the collecting duct epithelium and mice with specific deletion of *KLF5* in the collecting duct exerted enhanced interstitial fibrosis after unilateral ureteral obstruction (UUO).^24^ Chen *et al.* reported that increasing the matrix stiffness in cultured mouse proximal tubular epithelium cells (mPTECs) could up-regulate KLF5 expression, which promoted mPTECs proliferation.^25^ These data indicate that KLF5 involves in regulation of renal fibrosis progression under inflammation conditions. It is very interesting to analyze whether KLF5 has a functional role in ccRCC tumorigenesis and progression. Hence, we analyze online database, clinical patient samples and multiple ccRCC cell lines to uncover the potential role of KLF5 in ccRCC.

## Results

### KLF5 is significantly downregulated in ccRCC

**Figure 1:**
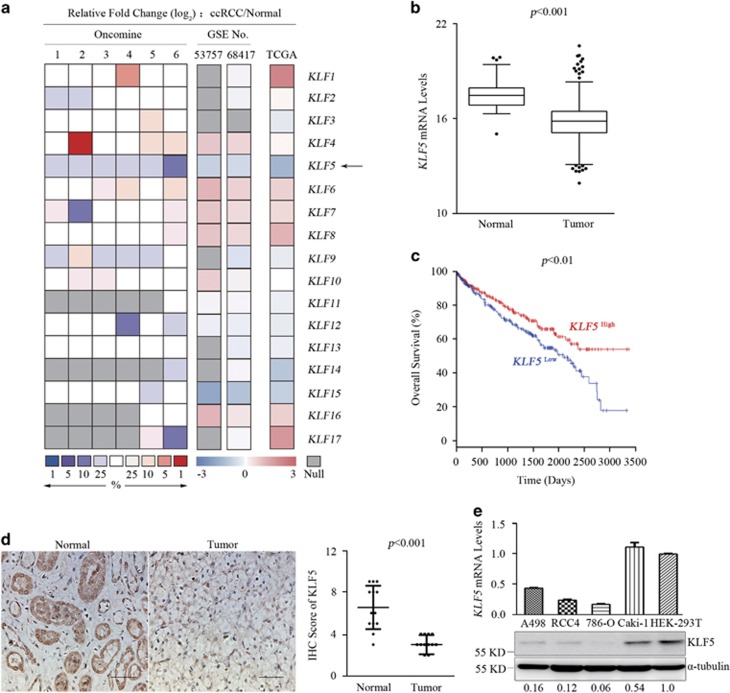
KLF5 expression is suppressed in ccRCC patients. (**a**) The heatmap showing the fold changes of mRNA expression levels of KLF family members compared ccRCC patients with normal people from the Oncomine, GEO and TCGA KIRC data sets. Arrow indicates relative change of KLF5. Oncomine data sets include: 1, Beroukhim renal; 2, Cutcliffe renal; 3, Gumz renal; 4, Jones renal; 5, Lenburg renal and 6, Yusenko renal. GSE53757 and GSE68417 are numbers of GEO data sets. (**b**) Boxplot of mRNA levels of *KLF5* in tissues of normal people (*n*=72) and ccRCC patients (*n*=531) from TCGA KIRC data sets. Two-tailed Student’s *t*-test, *P*<0.001. (**c**) Kaplan–Meier analysis of overall survival of ccRCC patients (*n*=531) segregated by low or high expression of *KLF5* from TCGA KIRC data sets. Log-rank test, *P*<0.01. (**d**) Representative IHC staining images (left) and IRS scores (right) of KLF5 expression of tumor or adjacent normal tissues from ccRCC patients (*n*=13). Scale bar, 50 *μ*m. Student’s *t*-test, *P*<0.001. (**e**) qPCR (up) and western blots (down) for the mRNA and protein levels of KLF5 of indicated ccRCC and HEK-293T cells. Expression levels of *KLF5* in ccRCC cells were normalized to that in HEK-293T cells

To explore whether members of KLF family involve in tumorigenesis of ccRCC, the expression levels of KLF1-17 genes were analyzed in Oncomine, GEO and TCGA KIRC data sets, respectively. Compared with normal people, it was particularly noteworthy that KLF5 was significantly and consistently inhibited in ccRCC among the KLF genes across the three data sets analyses (Figures 1a and b; Supplementary Figure 1). Further analysis of TCGA data sets revealed that higher expression level of KLF5 was associated with better prognostic outcome (Figure 1c). Overall survival rate of ccRCC patients with high KLF5 expression was significantly higher than patients with low KLF5 expression, especially after about 7 years (2500 days). These analyses indicated that KLF5 might be a tumor suppressor in ccRCC. To confirm these findings, protein levels of KLF5 were detected by immunohistochemistry (IHC) in clinical ccRCC tumors and adjacent normal tissues from Ren-Ji Hospital affiliated to Shanghai Jiao Tong University School of Medicine, and the related clinical information of these patients were shown (Supplementary Table 1). Protein levels of KLF5 were dramatically reduced in tumor areas than in adjacent normal renal tubule tissues (Figure 1d). Moreover, KLF5 expression was tested in different ccRCC cell lines and immortal embryonic kidney HEK-293T cells. KLF5 expression was distinctly inhibited in ccRCC cell lines compared with that in HEK-293T (Figure 1e). These results suggested that KLF5 might negatively influence the process of ccRCC.

### Hypermethylation suppresses KLF5 expression in ccRCC

Subsequently, we investigated how KLF5 was downregulated in ccRCC. It is well known that inactivation of the tumor suppressor gene Von Hippel-Lindau (*VHL*), including deletion, mutation and hypermethylation, is an archetypical tumor-initiating event in ccRCC, which leads to constitutive activation HIF-α that promotes tumorigenesis.^26^ To investigate whether *VHL* loss resulted in KLF5 suppression in ccRCC, VHL expression was detected in ccRCCs and HEK-293T cells. VHL could be detected in Caki-1 and HEK-293T cells, but not in 786-O, RCC4 and A498 cells (Supplementary Figure 2A). A ShRNA specifically against VHL was stably infected into VHL-expressed Caki-1 and HEK-293T cells, separately. Meanwhile, VHL was ectopically expressed in VHL-null 786-O, RCC4 and A498 cells. We found that KLF5 expression was inhibited no matter overexpression or inhibition VHL expression (Supplementary Figures 2B and C), which suggested that KLF5 suppression was not associated with VHL deficiency in ccRCC cells.

**Figure 2:**
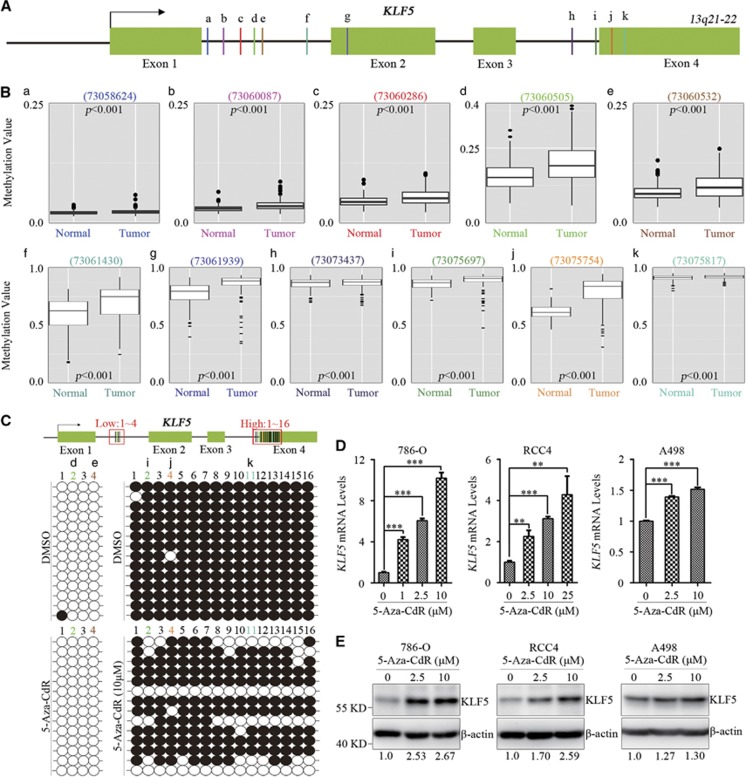
Hypermethylation suppresses *KLF5* expression in ccRCC. (**A**) A diagram represents methylation levels-altered CpG loci in *KLF5* gene of TCGA KIRC methylation array. Green box, arrow and lines indicates exons of *KLF5* gene, transcriptional start site and methylated loci (**a**–**k**) separately. (**B**) Methylation values of each indicated locus (**a**–**k**) in (**A**) were shown. Wilcoxon signed-rank test, *P*<0.001. (**C**) 786-O cells were treated with DMSO or 5-Aza-CdR (10 *μ*M), then bisulfite sequencing (down) was utilized to measure the methylation levels of the diagram indicated loci (up) in low (1–4) and high (1–16)-methylated areas. Open circles (○), unmethylated cytosine; closed circles (•), methylated cytosine. Sites 2/4 (d/e) in low-methylated area (1–4) and sites 2/4/11 (**i**/**j**/**k**) in high-methylated area (1–16) were known methylated loci from TCGA analyses. (**D**, **E**) qPCR and western blots for mRNA (**D**) and protein (**E**) level of KLF5 in indicated ccRCC cell lines with or without 5-Aza-CdR treatment. *P<0.05; **P<0.01; ***P<0.001 by two-tailed Student’s *t*-test. All bar graphs are plotted as mean±S.D. Expression level of KLF5 was normalized to its internal control, which in 5-Aza-CdR-treated ccRCC cells were compared with that in non-treated cells

DNA hypermethylation is a common mechanism for deregulation of tumor suppressor genes. Then, methylation alterations of CpG loci in *KLF5* gene were analyzed on DNA methylation array of TCGA KIRC data sets. Among the detected methylated loci in *KLF5* gene, the methylation level of eleven methylated loci (a-k) increased in ccRCC patients compared with normal people (Figures 2A and B). Five of these methylated loci located in low-methylated area whose methylation value ≤0.3 (Figures 2B, a–e) and the other six loci located in high-methylated area whose methylation value >0.7 (Figures 2B, f–k). To validate our findings in TCGA KIRC methylation array, methylation levels of genomic DNA of 786-O cells were analyzed. 786-O cells were treated with or without 5-Aza-CdR, a DNA methylation inhibitor which has been used in clinical cancer therapy. Bisulfite sequencing was then applied to examine the methylation levels of selected methylated loci in *KLF5* gene, of which two loci (Figures 2B, d) located at low-methylated area and three loci located at high-methylated area (Figures 2B, i–k) in TCGA methylation analyses. Consistent with TCGA results, methylation levels of five chosen methylated loci (Figures 2B, d) in low and high-methylated area were verified (Figure 2C), and 5-Aza-CdR treatment decreased methylation levels in high-methylated area of 786-O cells. Intriguingly, another fifteen CpG dinucleotides, including two (1/3) in low-methylated area and thirteen (1/3/5/6/7/8/9/10/12/13/14/15/16) in high-methylated area, were confirmed as new methylated loci that not reported in TCGA analyses (Figure 2C). These data suggested that *KLF5* gene was hypermethylated in ccRCC cells. To further investigate the correlation between hypermethylation and KLF5 expression, 786-O, RCC4 and A498 cells were treated with different concentrations of 5-Aza-CdR. The results demonstrated that both mRNA and protein level of KLF5 in these ccRCC cells were significantly and dose-dependently upregulated by 5-Aza-CdR (Figures 2D and E). Increasing levels in A498 cells were not as greatly as in 786-O and RCC4 cells, which might due to higher endogenous expression level of KLF5 in A498 cells than that in 786-O and RCC4 cells. These results suggested hypermethylation might contribute to the downregulation of KLF5 in ccRCC.

### Hypermethylation of KLF5 is mainly maintained by DNMT1 in ccRCC

**Figure 3:**
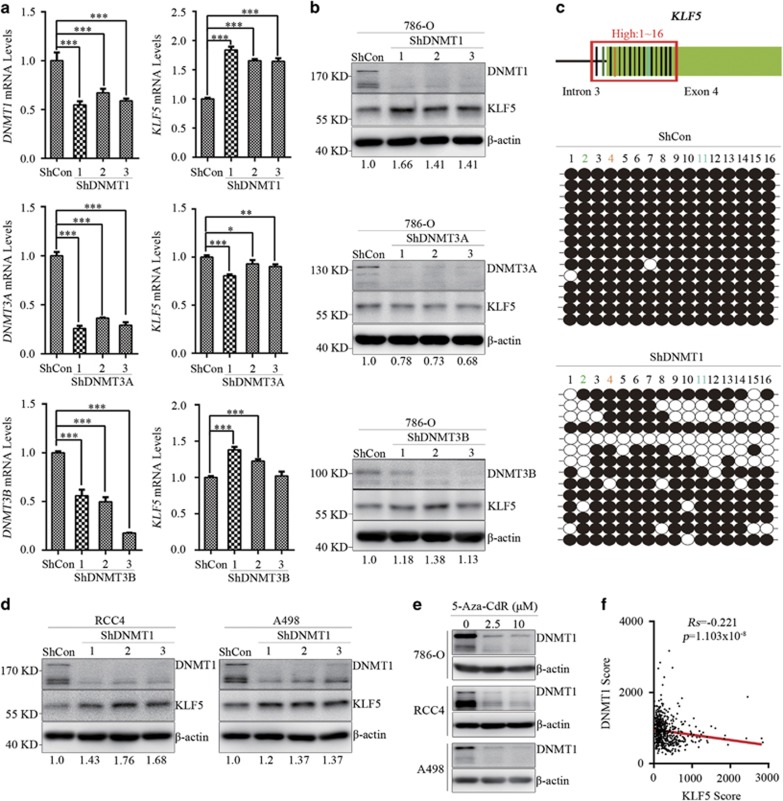
Hypermethylation of *KLF5* gene is mainly maintained by DNMT1. (**a**,**b**) 786-O cells were infected with ShDNMTs or ShControl (ShCon) lentivirus, then qPCR (**a**) and western blots (**b**) were applied to detect mRNA and protein levels of KLF5 and DNMTs. (**c**) Bisulfite sequencing (down) was applied to analyze the methylation levels of diagram-indicated loci (up) in high-methylated areas (1–16). Open circles (○), unmethylated cytosine; closed circles (•), methylated cytosine. (**d**) Western blots were utilized to detect expression of KLF5 in ShDNMT1 or ShCon-expressing RCC4 or A498 cells. Quantity One software was used to normalize KLF5 expression. Expression level of KLF5 was normalized to its internal control, and relative expression of KLF5 in ShDNMTs-expressed ccRCC cells were compared with that in ShCon-expressed cells. (**e**) Western blots for expression of DNMT1 in indicated ccRCC cell lines with or without 5-Aza-CdR treatment. (**f**) Scatter plots for the inverse correlation of KLF5 with DNMT1 expression in ccRCC patients (*n*=652) from online TumourProfile database. Spearman rank correlation test, *Rs*=Spearman rank correlation coefficient. All bar graphs are plotted as mean±S.D. *P<0.05; **P<0.01; ***P<0.001 by two-tailed Student’s *t*-test

DNA methylation is facilitated by three active enzymes, namely DNMT1, DNMT3A and DNMT3B. To determine which DNMTs contributed to *KLF5* hypermethylation in ccRCC, ShRNAs specifically against DNMT1, 3A and 3B were stably infected into 786-O, RCC4 and A498 cells separately. Compared with control ShRNA (ShCon) expressing 786-O cells, DNMT1 knockdown significantly increased KLF5 expression both in mRNA and protein level, while DNMT3A knockdown had no effect on KLF5 expression and DNMT3B knockdown slightly increased KLF5 expression (Figures 3a and b). Whether DNMT1 could influence methylation levels of *KLF5* was then examined. Genomic DNA of ShCon and ShDNMT1 expressing 786-O cells were extracted and bisulfite sequencing was used to examine the methylation levels of high-methylated area as described in Figure 2C. Compared with ShCon-expressing 786-O cells, knockdown of DNMT1 decreased *KLF5* methylation levels in high-methylated area (Figure 3c). This indicated that hypermethylation of *KLF5*, which suppressed its expression, was mainly maintained by DNMT1 in ccRCC. Consistent with the impact of DNMT1 on KLF5 expression in 786-O cells, negative correlation of DNMT1 on KLF5 expression were also evident in RCC4 and A498 cells (Figure 3d; Supplementary Figure 3A). It has been illustrated that 5-Aza-CdR is a DNA methylation inhibitor which leads to ubiquitin-dependent proteasome degradation of DNMT1.^27, 28^ To investigate whether 5-Aza-CdR-induced DNMT1 degradation contributed to KLF5 upregulation in ccRCC, DNMTs expression were detected in 786-O, RCC4 and A498 cells with or without 5-Aza-CdR treatment. 5-Aza-CdR treatment significantly decreased DNMT1 protein level rather than DNM3A and DNMT3B levels in ccRCC cells (Figure 3e; Supplementary Figure 3B). It has been described previously that DNMTs were overexpressed in ccRCC patients. Then, DNMTs expression were analyzed in TCGA KIRC data sets. Indeed, all of these DNMTs were highly expressed in ccRCC patients compared with normal people (Supplementary Figures 3C and D). Furthermore, the correlations between KLF5 and DNMTs expression were analyzed in 656 ccRCC samples from online TumourProfile database. Spearman rank correlation coefficient (*Rs*) of these samples showed the inverse correlation between KLF5 and DNMT1 expression in ccRCC (Figure 3f). Meanwhile, KLF5 had weaker correlation with DNMT3B expression than with DNMT1 but had no correlation with DNMT3A (Supplementary Figure 3E), which consistent with our *in vitro* results. In brief, our findings demonstrated that hypermethylation of KLF5 was mainly maintained by DNMT1.

### KLF5 inhibits ccRCC cell proliferation and migration/invasion *in vitro*

**Figure 4:**
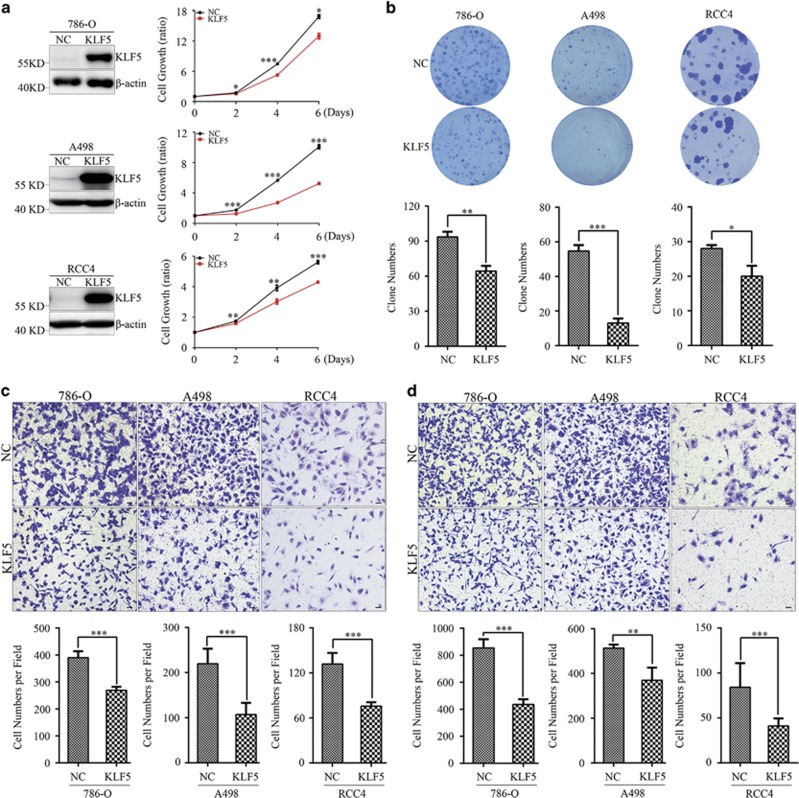
KLF5 inhibits ccRCC cell growth and migration/invasion *in vitro*. 786-O, A498 and RCC4 cells were stably infected with control (NC) or KLF5 lentivirus. (**a**) Western blots for KLF5 expression (left) and CCK8 analysis for proliferation (right) of indicated cells. (**b**) Representative colony formation of NC or KLF5-transduced 786-O, A498 and RCC4 cells (up) and statistical analyses of colony numbers (down). (**c**,**d**) Representative images of transwell migration (**c**) or invasion (**d**) assays of NC or KLF5-transduced 786-O, A498 and RCC4 cells (up) and statistical analyses of migrated or invaded cell numbers were shown (down). All experiments were repeated at least three times with triplicate samples. All bar graphs are plotted as mean±S.D. *P*-values are calculated between linked groups. *P<0.05; **P<0.01; ***P<0.001

Abundant evidences have demonstrated critical roles of KLF5 in regulating cell proliferation and migration/invasion in various cancers. Herein, we showed KLF5 expression was repressed by hypermethylation. This indicated KLF5 might be a tumor suppressor in ccRCC. To investigated the functional roles of KLF5 in ccRCC, KLF5 was overexpressed (OE) in 786-O, A498 and RCC4 ccRCC cell lines respectively. Ectopic expression of KLF5 effectively inhibited cell growth of these three cells (Figure 4a). Furthermore, colony formation ability of cells overexpressing KLF5 was markedly decreased in all of these three cell lines compared with related control cells (Figure 4b). Next, roles of KLF5 in ccRCC cells migration and invasion were evaluated. KLF5 overexpression could not only distinctly suppress transwell migration ability of 786-O, A498 and RCC4 cells (Figure 4c), but also restrain their invasion capacity (Figure 4d). Taken together, these data suggested KLF5 negatively associated with cell proliferation and migration/invasion in ccRCC cells.

### KLF5 expression suppresses ccRCC xenograft growth

**Figure 5:**
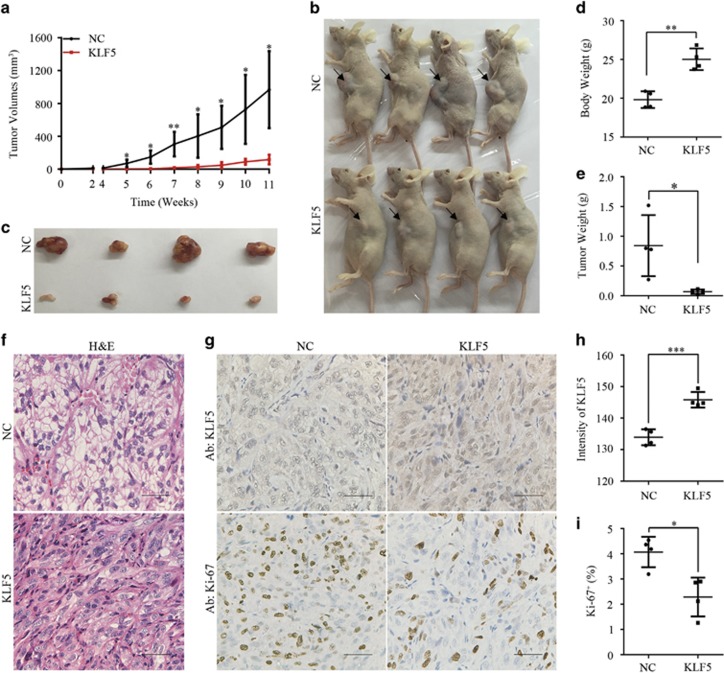
KLF5 expression inhibits ccRCC xenograft growth *in vivo*. NC or KLF5-transduced 786-O cells were inoculated subcutaneously into nude mice. (**a**) Tumor volumes were measured every week and growth curves of two groups were shown. (**b**–**e**) Images of euthanized mice (**b**) and excised tumors (**c**) were shown. Bodyweight with tumor (**d**) and tumor weight alone (**e**) were measured. (**f**–**i**) Representative images of H&E staining (**f**) or IHC staining of KLF5 or Ki-67 (**g**) of these tumors were shown. Scale bar, 50 *μ*m. Image J software was used to quantify the intensity of KLF5 (**h**) or Ki-67 positive cells (**i**) between NC and KLF5-transduced 786-O subcutaneous tumors. All bar graphs are plotted as mean±S.D. *P*-values are calculated between linked groups. *P<0.05; **P<0.01; ***P<0.001

Given that ectopic KLF5 expression inhibited ccRCC cell growth *in vitro*, whether KLF5 also affected tumor growth *in vivo* was further investigated. To this end, 786-O cells expressing KLF5 or control vector (NC) were subcutaneously injected in flanks of BALB/c nude mice. Consistent with the anti-proliferation effects in ccRCC cell lines, tumor growth of 786-O xenograft was significantly impaired accompanied with KLF5 expression (Figures 5a–c). Compared with mice burdened 786-O-NC xenograft, mice bearing 786-O-KLF5-expressing tumor had higher bodyweight and dramatically smaller tumor volume and tumor weight (Figures 5d and e). Furthermore, the histological morphology of ccRCC, which the malignant cells were surrounded by dense vascular endothelial cells and characterized with clear cytoplasm arranged in nests or acinar structures, was apparently improved in xenograft overexpressing KLF5 (Figure 5f). Immunohistochemistry (IHC) staining showed that high KLF5 staining was correlated with weak Ki-67 staining, and vice versa (Figures 5g–i). Together, these findings strongly indicated ectopic expression of KLF5 could significantly inhibited ccRCC growth *in vivo*.

### KLF5 expression inhibits ccRCC tumor metastasis

**Figure 6:**
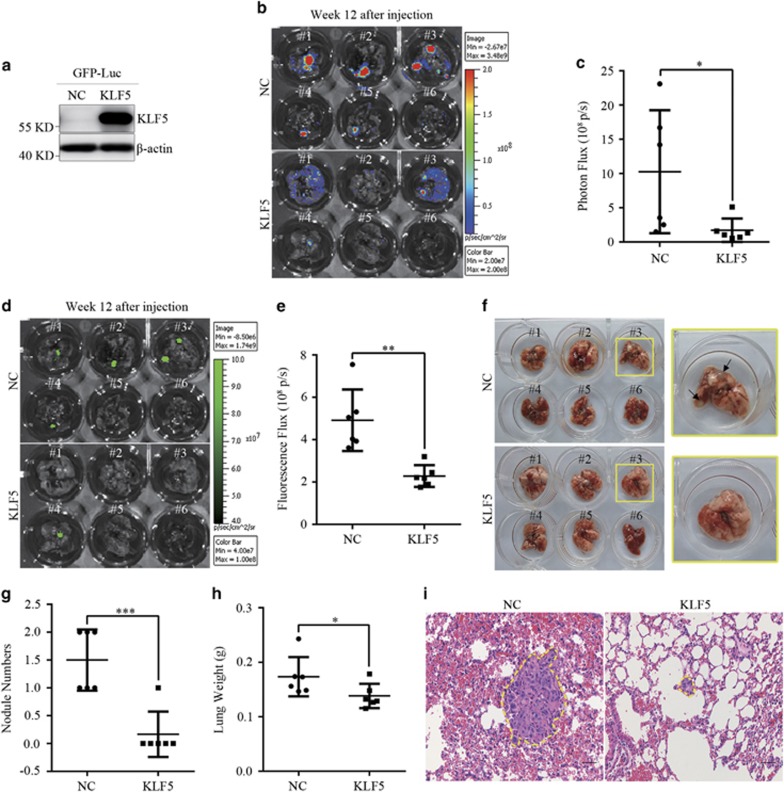
KLF5 expression suppresses ccRCC metastasis *in vivo*. GFP-luciferase (GFP-Luc) lentivirus was infected into NC and KLF5-transduced 786-O cells. These cells were then intravenously injected into nude mice. (**a**) Western blots were applied to detect expression of KLF5 in GFP-Luc cells. (**b**–**i**) Twelve weeks after injection, these mice were euthanized, and BLI images (**b**) and quantification of photon fluxes (**c**) or fluorescence images (**d**) and quantification of fluorescence fluxes (**e**) or images (**f**) of lungs were shown and compared. Black arrows in (**f**) indicated the metastatic nodules. Images at right are magnified view of yellow line circled images at left. The number of macroscopic lung metastasis nodules (**g**) and lung weight (**h**) were counted and compared. (**i**) Representative images of H&E staining of lung metastasis nodules (rounded by yellow lines) were shown. Scale bar, 50 *μ*m. All bar graphs are plotted as mean±S.D. *P<0.05; **P<0.01; ***P<0.001 by two-tailed Student’s *t*-test

Metastasis, a major cause of most cancer-related deaths, is a feature of malignant tumors.^29^ In Figures 4c and d, we showed that ectopic KLF5 expression inhibited ccRCC cell lines migration/invasion *in vitro*. To assess whether KLF5 could regulate tumor metastasis *in vivo*, 786-O control and OE KLF5 cells were infected with GFP-Luc separately (Figure 6a) and then retro-orbital venous plexus injected into nude mice. At week 12, all mice were euthanized and bioluminescence imaging (BLI) signal intensity of lungs from both group were detected. As displayed in Figures 6b and c, photon fluxes of lungs with KLF5-expressing tumor nodules decreased compared with that in control group. Consistently, fluorescence fluxes of lungs bearing KLF5-expressing tumors also decreased obviously (Figures 6d and e). Further histological analyses indicated KLF5 expression reduced the number of metastatic nodules and lung weight, which was consistent with the BLI signal intensity detected *in vivo* (Figures 6f–h). Representative hematoxylin-eosin (H&E) staining exhibited KLF5 expression inhibited the formation of metastatic colonies in lungs (Figure 6i). Therefore, these studies demonstrated KLF5 could effectively suppress the metastatic properties of ccRCC *in vivo*.

### 5-Aza-CdR inhibits ccRCC cell proliferation through demethylation of KLF5

**Figure 7:**
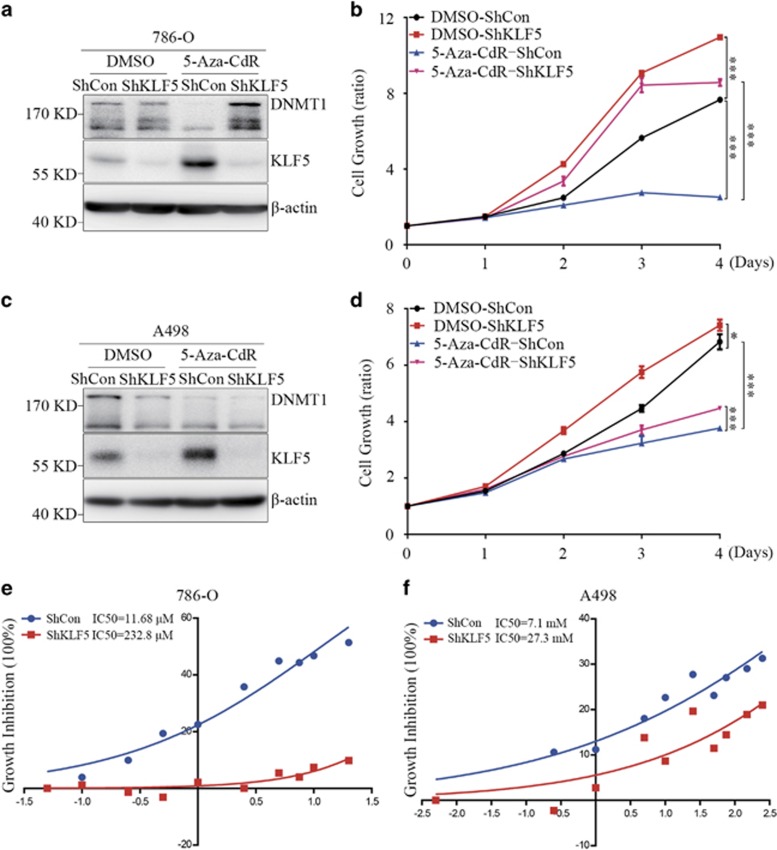
5-Aza-CdR inhibits ccRCC cell proliferation partially by demethylation of *KLF5*. (**a**–**d**) ShControl (ShCon) or ShKLF5 were stably expressed in 786-O or A498 cells, then cells were treated with or without 5-Aza-CdR (1 *μ*M for 786-O cells and 10 *μ*M for A498 cells) for 4 days. Western blots for DNMT1 and KLF5 expression (**a** and **c**) and CCK8 assay for cell proliferation (**b** and **d**) of these cells were shown. Cell growth curves were separately compared between ShCon with and without 5-Aza-CdR treatment or between ShCon and ShKLF5 with and without 5-Aza-CdR treatment. The statistical significance was indicated by *P*-value. (**e**–**f**) 786-O (**e**) or A498 cells (**f**) expressing ShCon and ShKLF5 were treated with different concentrations of 5-Aza-CdR and IC50 were calculated by GraphPad Prism 6.0 software. All experiments were repeated at least three times with triplicate samples. All bar graphs are plotted as mean±S.D. *P<0.05; **P<0.01; ***P<0.001 by two-tailed Student’s *t*-test

Since hypermethylation of *KLF5* suppressed its expression and ectopic KLF5 could inhibit ccRCC growth both *in vitro* and *in vivo*, it is very interesting to evaluate whether upregulating endogenous KLF5 expression by demethylation of *KLF5* gene could inhibit cell growth. To this purpose, 786-O and A498 cells were stably infected with a ShRNA specifically against KLF5 (Figure 7a). Then cells expressing ShControl or ShKLF5 were treated with or without 5-Aza-CdR for 4 days, and proliferation of cells was measured every day. Accompanied by the upregulation of KLF5, 5-Aza-CdR treatment significantly inhibited cell growth of 786-O cells, and knockdown KLF5 by ShRNA could dramatically attenuate 5-Aza-CdR-induced growth inhibition (Figure 7b). Similar results also could be observed in A498 cells (Figures 7c and d), even though the effect was not as significant as in 786-O cells. These results indicated that KLF5 mediated 5-Aza-CdR-induced growth inhibition of ccRCC cells. Furthermore, ShKLF5 or ShCon-expressing 786-O or A498 cells were exposed to different doses of 5-Aza-CdR. As showed in Figures 7e and f, cells expressing ShKLF5 endowed higher concentration of 5-Aza-CdR treatment than those ShCon-expressing ccRCC cells, which suggested that KLF5 expression contributed to the growth inhibition of 5-Aza-CdR.

## Discussion

The roles of KLF5 in tumorigenesis are due to its cellular and genetic context. In this study, our data evidence that KLF5 is a tumor suppressor in ccRCC based on the following facts: first, KLF5 expression is inhibited in ccRCC patients no matter through database analysis or clinical patient samples histochemical staining, and patients with high expression of KLF5 have better prognostic outcome; second, KLF5 expression is suppressed by DNMT1-maintained genomic hypermethylation, and ectopic expression of KLF5 inhibits ccRCC cells proliferation and migration/invasion *in vitro* and xenograft growth and metastasis *in vivo*; last but not least, DNMTs’ inhibitor 5-Aza-CdR can restore KLF5 expression and suppress ccRCC cell growth, while knockdown KLF5 can decrease drug sensitivity to 5-Aza-CdR and abolish 5-Aza-CdR-induced cell growth inhibition.

In mammals, DNA methylation is present predominantly in the context of CpG dinucleotides and is involved in regulation of chromatin structure and gene expression. Hypermethylation of promoter or enhancer can result in inactivation of important tumor suppressor genes whereas hypomethylation of genomic DNA is associated with chromosomal instability and tumorigenesis.^30, 31, 32^ DNA methylation is mediated by a family of enzymes named DNMTs. To date, the known DNMTs are DNMT1, DNMT2, DNMT3A, DNMT3B and DNMT3L. Methylation can be *de novo* methylation of CpG dinucleotides on totally unmethylated DNA strands or maintenance methylation of CpG dinucleotides on DNA that one strand has been methylated. DNMT1 has both *de novo* and maintenance methyltransferase activity, while DNMT3A and DNMT3B are powerful *de novo* methyltransferases. DNMT2 is shown to methylate RNA and DNMT3L does not have methyltransferase activity.^8^ Previous studies have showed hypermethylation of *KLF5* in intron 1 that downregulated KLF5 expression might correlated with DNMT3A mutation in AML.^21, 33, 34^ In this study, we found that KLF5 expression was also suppressed by hypermethylation, but the methylated loci reported in AML, including that in proximal promoter (−529 to −318 bp from transcription start site (TSS)) and in intron 1 (+1284 to +1571 bp from TSS), could not be found in ccRCC. Although DNMT3A might contribute to the hypermethylation of *KLF5* in AML, knockdown of DNMT3A could not restore KLF5 expression in ccRCC cells, but inhibition of DNMT1 could. Online data sets analyses indicated that DNMT3B expression was slightly correlated with KLF5 expression in ccRCC. When knockdown DNMT3B, KLF5 expression was upregulated weakly. Whether DNMT3B contributes to the *de novo* methylation of KLF5 in ccRCC remains to be elucidated. The presence of increased methylation level of eleven methylated loci of *KLF5* in ccRCC patients indicated dysregulation of methylation at *KLF5* genome by TCGA analyses. Surprisingly, we first observed that hypermethylated loci of *KLF5* located at proximal exon 4 in ccRCC. When treated with 5-Aza-CdR or knockdown DNMT1 expression, methylation levels of ccRCC cells at proximal exon 4 area were decreased. These findings indicated that hypermethylated loci at proximal exon 4 area, which were far away from *KLF5* TSS, could affect KLF5 expression in ccRCC. The mechanisms why DNA hypermethytion is present at proximal exon 4 in ccRCC and how DNMT1 regulates *KLF5* hypermethylation remain to be explored. The current view of DNA methylation indicates that the correlation between CpG methylation and gene expression depends on genomic context. CpGs locate both in gene regulatory regions, which are less than 2 kb away from the TSS, and in the gene bodies, which are more than 2 kb away from the TSS. Methylation of CpGs at gene regulatory regions is negatively correlated with gene expression, while methylation of CpGs at gene bodies be either positively or negatively correlated with gene expression. When CpGs locate at gene bodies but do not reside in a CpG island (CGI), methylation of CpGs is positively correlated with gene expression. When CpGs locate at gene bodies and also reside in a CGI, methylation is negatively correlated with gene expression. What’s more, the CpG-rich regions in the gene bodies whose methylation is negatively correlated with gene expression might be intragenic enhancers.^35^ Whether the methylation loci we found is an intragenic enhancer of *KLF5* gene remains to be elucidated.

Due to various cellular and genetic context, KLF5 has exerted pro- or anti-tumorigenic function in different types of tumors. In our study, KLF5 functions as a tumor suppressor in ccRCC. It has been found that acetylation of KLF5 influenced its roles in tumor and interruption KLF5 acetylation could reverse its role from a tumor suppressor to a tumor promoter.^36, 37^ Whether acetylation of KLF5 involves in the tumorigenesis of ccRCC remains to be investigated. Xing *et al.* demonstrated that *KLF5* deletion in *PTEN*-null mice upregulated epidermal growth factor (EGF) and its downstream signaling molecules AKT and ERK and initiated luminal-type mouse prostate tumors.^38^ It has been reported that tumor suppressor gene *PTEN* was deleted or mutated during ccRCC carcinogenesis.^7, 39^ KLF5 also could inhibit the activation of ERK in ccRCC (data not shown). These findings indicated that KLF5-ERK axis might regulate cell growth in ccRCC. Zhang *et al.* reported that KLF5 could inhibit epithelial-mesenchymal transition (EMT) through activation microRNA200 expression.^40^ Whether KLF5 inhibits ccRCC metastasis mediated by microRNAs deserves to be deeply explored. The underlying mechanisms that how KLF5 has a tumor suppressor role in ccRCC need to be further studied.

Despite the epigenetic regulation of KLF5 in ccRCC, post-translational regulation of KLF5, especially protein stability, was also been considered. It has been reported that KLF5 was degraded by ubiquitin-proteasome pathway (UPP). At present, several E3 ligases, including WWP1,^41^ EFP,^42^ Smurf2^43^ and FBW7,^44^ were discovered to ubiquitinate KLF5 which led to its degradation. MG132, a 26 S proteasome inhibitor, was used to treat ccRCC cells. KLF5 protein was stabilized by MG132 treatment, especially in A498 cells (Supplementary Figure 4). Thus, protein instability was also contributed to the low level of KLF5 in ccRCC cells. In addition, KLF5 can be stabilized by the deubiquitinase (DUB) BAP1,^45^ and *BAP1* gene is inactivated in 15% ccRCC and defines a new class of ccRCC.^46^ Inactivation of deubiquitinases and over-activation of E3 ligases might be post-translational mechanism for the suppression of KLF5 in ccRCC.

In this study, we demonstrated that KLF5, as a tumor suppressor, was suppressed by DNMT1-maintained hypermethylation in ccRCC. DNMTs’ inhibitor 5-Aza-CdR could suppress ccRCC cell growth and induce KLF5 expression, and KLF5 mediated 5-Aza-CdR-induced growth inhibition. Collectively, our research highlights the potential of 5-Aza-CdR, a methylation inhibitor that can rescue KLF5 expression, as a therapeutic modality for the treatment of ccRCC.

## Materials and Methods

### Cell lines and reagents

Human ccRCC cell lines 786-O, RCC4, A498, Caki-1 and immortal embryonic kidney cell line HEK-293T were obtained from the cell bank of the Chinese Academy of Sciences (Shanghai, China). RCC4, A498 and HEK-293T cells were cultured in Dulbecco’s modified Eagle’s medium (Hyclone, Logan, UT, USA) with 10% fetal bovine serum (FBS, Sigma-Aldrich, St. Louis, MO, USA). 786-O and Caki-1 cells were maintained in RPMI 1640 (Hyclone) with 10% FBS. All cells were cultured in a 95% air and 5% CO_2_ humidified atmosphere at 37 °C. 5-Aza-CdR (Sigma-Aldrich, A3656) was dissolved in DMSO as stocking solution and diluted in sterile PBS before use.

### Patient cohort

Paired specimens of tumor and adjacent tissues of ccRCC patients (*n*=13), which were histopathologically diagnosed during 2015 and 2016, were obtained in Ren-Ji Hospital affiliated to Shanghai Jiao Tong University School of Medicine. All samples were primary tumors and untreated before surgery. Detailed information was described in the Supplementary Table 1. These studies were approved by the Medical Ethical Committee of Ren-Ji Hospital, and informed consent was obtained from all subjects or their relatives.

### Immunohistochemical staining

IHC was applied to detect the protein levels of KLF5 between ccRCC tumors and adjacent normal tissues with anti-KLF5 (Sigma-Aldrich, HPA040398) polyclonal antibody. IHC staining was performed according to the manufacturer’s protocol. All staining were blindly scored by two pathologists according to the intensity of the nucleus, cytoplasmic and/or membrane staining (no staining=0; weak staining=1, moderate staining=2, strong staining=3) and the area extent of stained cells (0%=0, 1–24%=1, 25–49%=2, 50–74%=3, 75–100%=4). The final immunoreactive score (IRS) was determined by multiplying the intensity score with the extent score of stained cells, ranging from 0 (the minimum score) to 12 (the maximum score). Final scores of KLF5 between thirteen pairs of ccRCC patients and normal adjacent tissues were calculated by GraphPad Prism 6.0 software. IHC staining of KLF5 and Ki-67 for 786-O xenograft were performed with anti-KLF5 and anti-Ki-67 (Abcam, Cambridge, MA, USA, ab16667) polyclonal antibody. The intensity of KLF5 and Ki-67 positive cells were quantified by Image J software.

### Plasmids, ShRNA design and viral infection

Human *KLF5* cDNA was cloned and inserted into pLVX-Puromycin lentiviral expression vector (Clontech, CA, USA). GV298-CMV-mU6-MSC-Cherry-Puromycin lentiviral plasmid expressing ShKLF5 and control plasmid were purchased from GENECHEM (Shanghai, China). The target sequence for ShKLF5 was shown in Supplementary Table 2. pGIRZ-hCMV-tGFP-Puromycin lentiviral plasmids against DNMTs DNMT1/3A/3B and control were obtained from department of biochemistry and molecular biology, Shanghai Jiao Tong University School of Medicine. The target sequences for ShDNMTs were shown in Supplementary Table 2. pBABE-puro-*VHL*-Flag and pSIREN-ShVHL plasmids were gifts from Prof. Li-Shun Wang (Central Hospital of Min Hang District, Shanghai, China). These plasmids were co-transfected with packaging plasmids including psPAX2 and pMD2G for lentivirus or VSVG and gag-pol for retrovirus into HEK-293T cells to produce lentivirus or retrovirus. Forty-eight hours after transfection, the viral supernatants were harvested, filtered through 0.45 *μ*M membrane (Merck-Millipore, MA, USA) and added respectively into 786-O, RCC4 and A498 cells incubated with the medium containing 0.8 *μ*g/ml polybrene (Santa Cruz Biotechnology, Texas, USA, sc-134220). Stably expressed cells were selected by 1 *μ*g/ml puromycin after viral infection for 48 h. Selection was stopped as soon as the non-infected control cells died off, and the media were replaced with normal growth media.

### Western blots

Western blots was performed as described before.^47^ Western blots images were acquired using the LAS-4000 CCD imaging system (Fujifilm, Japan). The following antibodies were used: Rabbit polyclonal antibodies against KLF5 (Proteintech Group, Rosemont, IL, USA, 21017-1-AP), VHL (Novus Biologicals, Littleton, CO, USA, NB100-485), DNMT1 (Cell Signaling Technology, Beverly, MA, USA, #5032), DNMT3A (Cell Signaling Technology, #D23G1), Mouse monoclonal antibody against DNMT3B (Santa Cruz Biotechnology, sc-376043), HRP-linked *β*-actin monoclonal antibody (Proteintech Group, HRP-60008) and HRP-linked *α*-tubulin polyclonal antibody (MBL International Corporation, MA, USA, PM0547). The protein levels were quantified by Quantity One software.

### Quantitative real-time RT-PCR

Total RNA was extracted by TriPure Isolation Reagent (Roche, Basel, Switzerland) and reverse transcription was carried out using M-MLV reverse transcriptase (Promega, Madison, WI, USA). QPCR of the target genes were carried out with Power SYBR Green PCR Master mix (Applied Biosystems, Foster City, CA, USA) using ABI PRISM 7900HT Fast system (Thermo Fisher Scientific, Waltham, MA, USA). Experiments were repeated at least three times with similar results. A list of qPCR primers could be found in Supplementary Table 3.

### Cell proliferation and colony formation assay

Cells proliferation was evaluated by the CCK8 assay (WST-8; Cell counting kit-8 from Dojindo, Japan). Briefly, 100 *μ*l cells were seeded into 96-well plates at a density of 2000 cells/well. WST-8 was added and absorbance readings at a wavelength of 450 nm were taken on Synergy H4 Hybrid Microplate Reader (BioTek, Winooski, VT, USA). For colony formation assay, 600 cells for RCC4 and 1000 cells for 786-O or A498 were plated in complete growth media and allowed to grow until visible colonies formed. Cell colonies were stained with 0.1% crystal violet and photographed. Total cell numbers were counted and calculated by GraphPad Prism 6.0 software (San Diego, CA, USA).

### Transwell migration and invasion assay

For transwell migration assay using 786-O, RCC4 and A498 cells, twenty-five thousand cells were plated on 8-*μ*M transwell filters (Corning, NY, USA). For transwell invasion assay, fifty thousand cells were seeded and meanwhile, inserts were coated on the inside with Matrigel (BD Biosciences, San Jose, CA, USA). Cells in insert chambers, which were cultured with no FBS, migrated towards lower compartment that containing medium with FBS. Non-migration and invasion cells were removed with a cotton swab. The remaining cells were stained with crystal violet and photographed. Cells in fifteen random fields were counted under microscope and calculated by GraphPad Prism 6.0 software.

### Animal experiments

Five million 786-O cells expressing KLF5 or control vector were subcutaneously injected into 6-week-old female BALB/c nude mice (*n*=4 for each group). Tumor was measured weekly and volume was calculated according to the following formula: volume= length × width × (width/2). At week 11, mice were euthanized and applied to further histological analysis. For lung metastasis assay, KLF5 or vector expressed 786-O cells were infected with GFP-Luc, then three million cells were injected into retro-orbital venous plexus of nude mice (*n*=6 for each group). For bioluminescence imaging (BLI), D-luciferin (YEASEN, Shanghai, China) was injected into anesthetized mice and bioluminescence images were captured (Xenogen IVIS, PerkinElmer, Waltham, MA, USA). At week 12, mice were euthanized and applied to further histological analysis. All measurements were performed blindly and all animals were manipulated and housed according to protocols approved by Shanghai Medical Experimental Animal Care Commission.

### Conventional bisulfite sequencing

For bisulfite sequencing, extracted genomic DNA was bisulfite converted according to the manufacturer’s instructions of an EZ DNA Methylation-Direct Kit (Zymo Research, CA, USA). Bisulfite-treated genomic DNA was subjected to PCR amplification. Primers used for detection methylation levels of *KLF5* gene in low-methylated and high-methylated area were shown in Supplementary Table 4. For further sequencing analysis, PCR products were purified with a Gel Extraction Kit (MACHEREY-NAGEL, Düren, Germany) and cloned into pMD19-T vectors (Takara, Kusatsu, Japan). Individual clones were sequenced and analyzed with original sequences by DNAMAN software.

### Cell viability assay and IC50 values

To evaluate drug sensitivity of ccRCC cells to 5-Aza-CdR, 786-O or A498 cells were seeded in 96-well plates at a density of 4000 cells/well for 786-O and 3000 cells/well for A498. Cells were then exposed to different concentrations of 5-Aza-CdR for 48 h. Then, CCK8 reagent was added and cell growth was measured by the absorbance at wavelength of 450 nm. The half-maximal inhibitory concentration (IC50) values were calculated by nonlinear regression analysis using the GraphPad Prism 6.0 software.

### RNA-sequencing and Microarray data sets analysis

Expression levels of *KLF5* were obtained from the Cancer Genome Atlas (TCGA), Oncomine and GEO respectively. TCGA clear cell kidney carcinoma (TCGA KIRC) data set portal (https://tcga-data.nci.nih.gov/tcga/) includes 72 normal people and 531 ccRCC patients. To segregate 531 patients according to high- or low-*KLF5* expression, the median expression was calculated. If *KLF5* expression was under the median value, patients were regarded as *KLF5*-low group, and *vice versa*. CpG DNA methylation array of TCGA KIRC data set was used to analyze methylation level of each *KLF5* CpG locus between KIRC patients and normal people. Six cohorts of ccRCC in Oncomine database (http://www.oncomine.org) were analyzed, and threshold was set as *P*<0.05 and fold change>2. GSE53757 and GSE68417 were selected from GEO data sets. GEO2R (https://www.ncbi.nlm.nih.gov/geo/geo2r/) interactive web tool was used to compare different *KLF5* expression between normal people and ccRCC patients. Normalized gene expression values of 652 ccRCC from the TumourProfile database (http://tumour.bjmu.edu.cn/), whose original data were obtained from GEO, were downloaded and analyzed.

### Statistical analysis

All the statistical analyses were evaluated using the GraphPad Prism 6.0 software. Each experiment was repeated at least three times. Data were presented as mean±S.D., and Student’s *t*-test (unpaired, two-tailed) was used to compare two groups of independent samples. Kaplan–Meier method was used to analyze overall survival (OS) and comparisons were analyzed by log-rank test. Statistical significance of Spearman rank correlation coefficient (*Rs*) was determined by Spearman rank correlation test. *P*<0.05 was considered to be statistically significant.
