# Supplementary material for: DNMT1-maintained hypermethylation of Krüppel-like factor 5 involves in the progression of clear cell renal cell carcinoma
Source: Cell Death Dis. 2017 Jul 27;8(7):e2952–. doi: 10.1038/cddis.2017.323 (PMC5550868; doi:10.1038/cddis.2017.323)
Supplement: Supplementary Information [file cddis2017323x1.docx]

**Supplemental Tables**

**Table S1**

| No. of patients | 13 |
| --- | --- |
| Age (Years+SD) | 62.31+13.34 |
| Sex (Male/Female) | 8/5 |
| Kidney cancer subtype | Renal clear cell carcinoma |
| Fuhrman grade |  |
| I | 3 |
| I-II | 1 |
| II | 6 |
| II-III | 2 |
| III | 1 |
| III-IV | 0 |
| IV | 0 |

**Table S1 related to Figure 1D. Clinical information of 13 pairs ccRCC patients.** The ccRCC patient specimens were collected in Ren-Ji Hospital affiliated to Shanghai Jiao Tong University School of Medicine. The clinical information, including age, sex, cancer subtype and Fuhrman grade, was shown in Table S1.

**Table S2. Target sequences used for ShRNA.**

| Gene | Target Sequence |
| --- | --- |
| *KLF5* | 5’-CTGCCAGTTAACTCACAAA-3’ |
| *DNMT1*-1 | 5’-TGGGAAGAAGAGTTACTAT-3’ |
| *DNMT1*-2 | 5’-CCAATGAGACTGACATCAA-3’ |
| *DNMT1*-3 | 5’-AGGAACTTTGTCTCCTTCA-3’ |
| *DNMT3A*-1 | 5’-ACGAGGTCAAACTCCATAA-3’ |
| *DNMT3A*-2 | 5’-AGGATAGCCAAGTTCAGCA-3’ |
| *DNMT3A*-3 | 5’-GCCTCAGAGCTATTACCCA-3’ |
| *DNMT3B*-1 | 5’-GCCCATTTGACTTGGTGAT-3’ |
| *DNMT3B*-2 | 5’-CGGCTCTTCTTCGAATTTT-3’ |
| *DNMT3B*-3 | 5’-AGAAGAGAAAATGTTGTAT-3’ |

**Table S3. Primers used for qPCR.**

| Gene | Forward Primer | Reverse Primer |
| --- | --- | --- |
| *KLF5* | CTTCCACAACAGGCCACTTACTT | AGAAGCAATTGTAGCAGCATAGGA |
| *DNMT1* | AAGGGAAGGGCAAGGGAAAAGG | AGAAAACACATCCAGGGTCCGCAG |
| *DNMT3A* | GATTGATGCCAAAGAAGTGTCAG | CATTCACAGTGGATGCCAAC |
| *DNMT3*B | AATGTGAATCCAGCCAGGAAAGGC | ACTGGATTACACTCCAGGAACCGT |
| *18s rRNA* | GTAACCCGTTGAACCCCATT | CCATCCAATCGGTAGTAGCG |

**Table S4. Primers used for methylation assay.**

| Area | Forward Primer | Reverse Primer |
| --- | --- | --- |
| Low | GGGTTTAGTTTAGTTTATTTTAAGATATTT | TTAGGATGTTTGTATTATAAGTAGGT |
| High | CCTCTCACAACAAAACCCTAAC | CTATCATTTAAAAAAACCCAAAAAAC |

**Supplemental Figures and Figure legends**


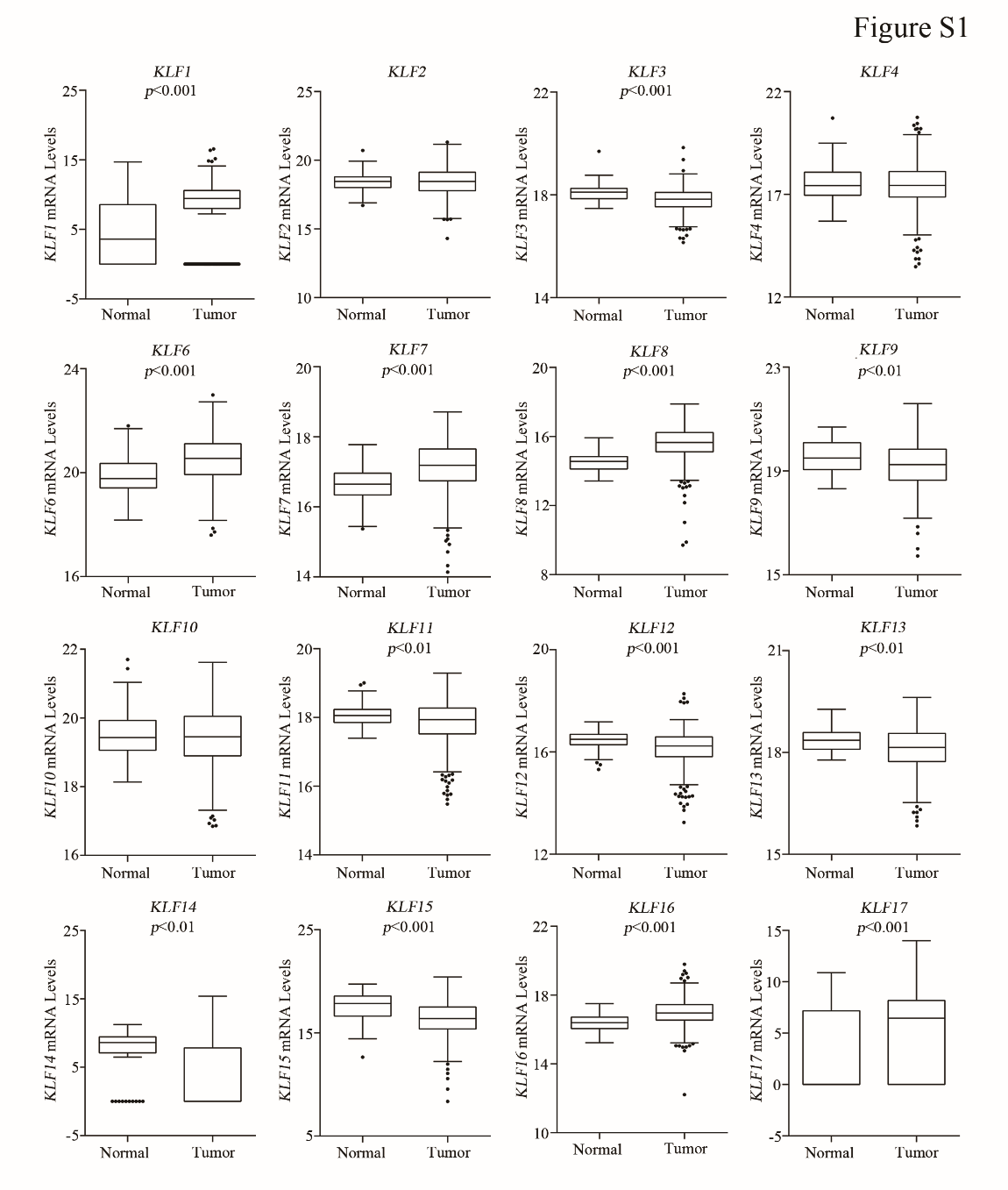


**Figure S1. The expression of *KLF* genes in TCGA KIRC datasets.**

RNA-sequencing data of the Cancer Genome Atlas (TCGA) clear cell kidney carcinoma (KIRC) datasets were downloaded and normalized, then expressions of *KLFs* in ccRCC (KIRC) patients were compared with normal people and statistical analyses were carried out by Student’s *t* test. Significant difference was indicated by *p*<0.01 and *p*<0.001.


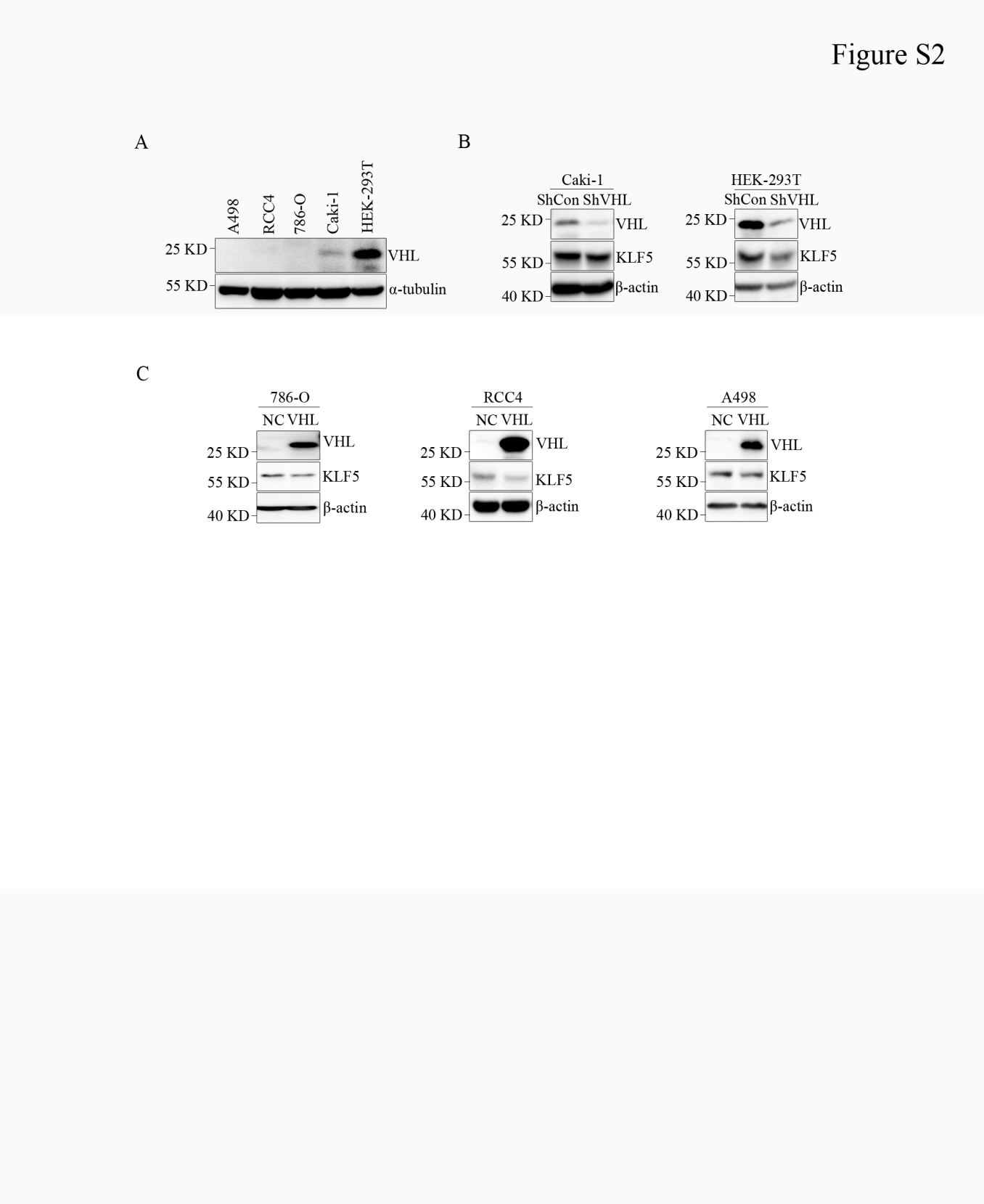


**Figure S2.** **VHL negatively regulates KLF5 expression in VHL-deficient ccRCC cell lines.**

(A) Western blots were applied to detect VHL expression in different ccRCC cell lines and HEK-293T cell line. (B) Caki-1 and HEK-293T cells were infected with ShVHL or ShControl (ShCon) retrovirus. Western blots were applied to detect VHL and KLF5 expression. (C) 786-O, RCC4 and A498 cells were infected with control (NC) or VHL lentivirus. Western blots were applied to detect VHL and KLF5 expression.


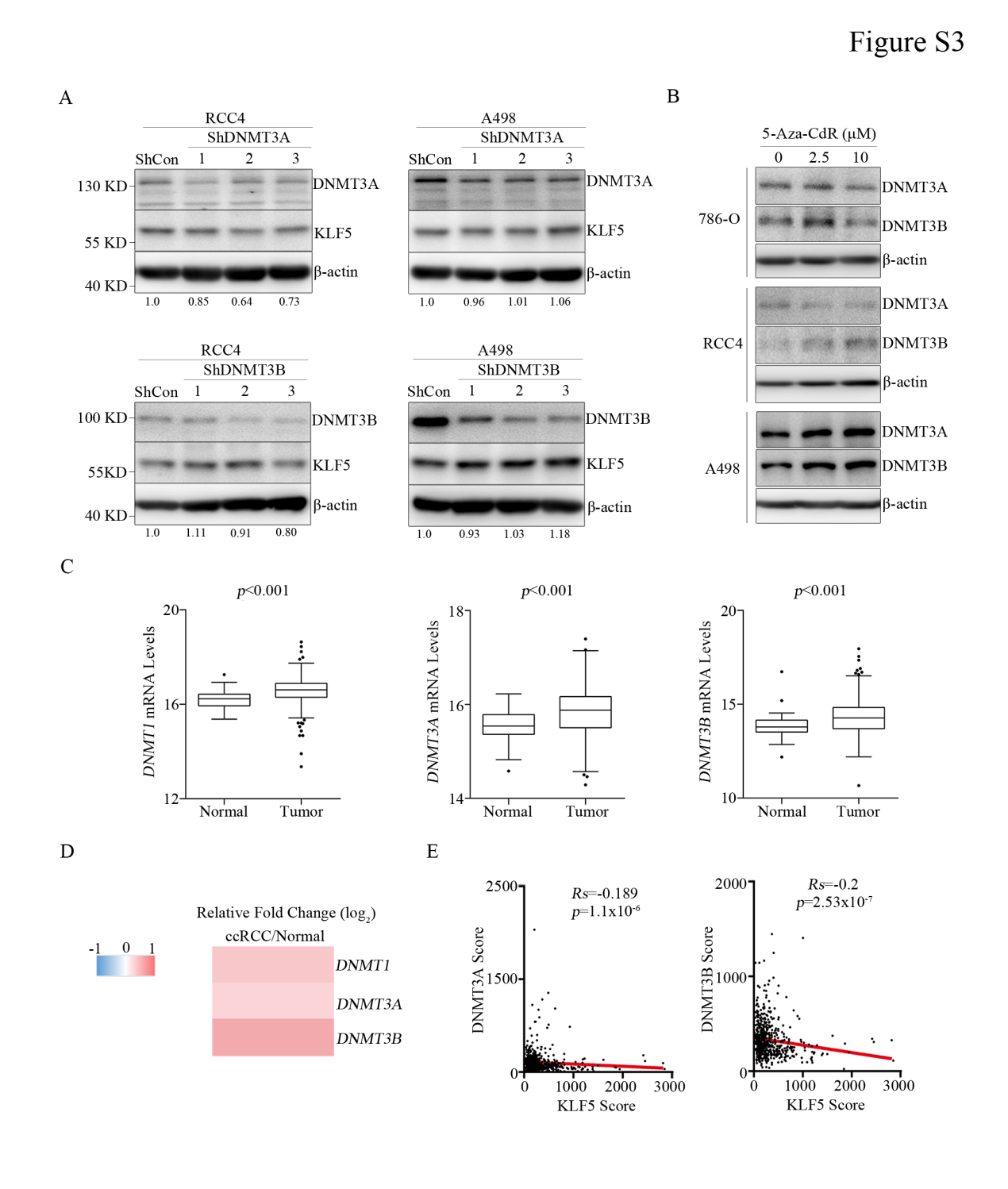


**Figure S3. Hypermathylation of *KLF5* gene loci is mainly maintained by DNMT1.**

(A) RCC4 or A498 cells were infected with ShDNMTs or ShControl (ShCon) lentivirus, western blots were applied to detect protein levels of KLF5 and DNMTs with β-actin as loading control. Quantity One software was applied to normalized KLF5 expression with internal control. (B) Western blots were applied to detect proteins levels of DNMT3A and DNMT3B in ccRCC cell lines with or without 5-Aza-CdR (10 μM) treatment. (C-D) Comparison of mRNA levels of DNMTs in normal people (n=72) and ccRCC patients (n=531) tissues from TCGA datasets (C) and heatmap of relative fold changes for DNMTs expression (D). Student’s *t* test, *p*<0.001. (E) Scatterplots showing the correlation of KLF5 with DNMT3A or DNMT3B expression in ccRCC (n=652) from TumourProfile database. Spearman rank correlation test, *Rs* = Spearman rank correlation coefficient.


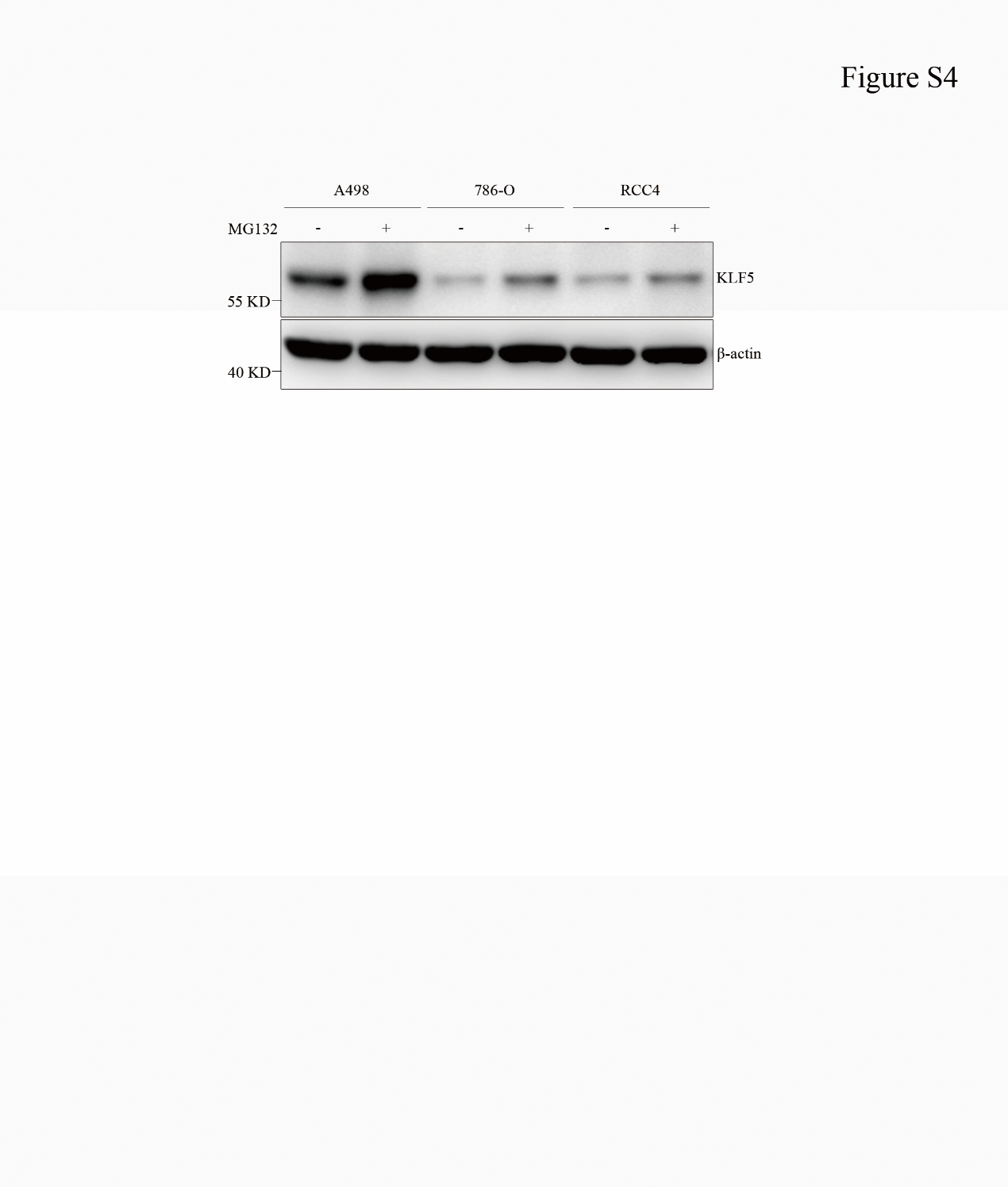


**Figure S4. Proteasome inhibitor MG132 could stabilize KLF5 protein in ccRCC cells.**

786-O, A498 and RCC4 cells were treated with or without proteasome inhibitor MG132 (20 μM) for 4 hours. Western blots were applied to detect expression of KLF5 with β-actin as loading control.
